# Proliferation Cycle Causes Age Dependent Mitochondrial Deficiencies and Contributes to the Aging of Stem Cells

**DOI:** 10.3390/genes8120397

**Published:** 2017-12-19

**Authors:** Qiuting Ren, Fan Zhang, Hong Xu

**Affiliations:** Laboratory of Molecular Genetics, National Heart, Lung, and Blood Institute, National Institutes of Health, Bethesda, MD 20892, USA; renqiuting@gmail.com (Q.R.); zhang.fan@nih.gov (F.Z.)

**Keywords:** aging, stem cell, mitochondria, mtDNA, telomere, DNA replication, DNA rearrangement, rolling cycle amplification

## Abstract

In addition to chronological aging, stem cells are also subject to proliferative aging during the adult life span. However, the consequences of proliferative cycle and their contributions to stem cells aging have not been well investigated. Using *Drosophila* female germ line stem cells as a model, we found that the replication cycle leads to the age dependent decline of female fecundity, and is a major factor causing developmental abnormalities in the progeny of old females. The proliferative aging does not cause telomere shortening, but causes an accumulation of mitochondrial DNA (mtDNA) mutations or rearrangements at the control region. We propose that damaging mutations on mtDNA caused by accumulation of proliferation cycles in aged stem cells may disrupt mitochondrial respiration chain and impair mtDNA replication and represent a conserved mechanism underlying stem cell aging.

## 1. Introduction

Besides the nuclear genome, a typical animal cell also has from 100 to 1000 copies of mitochondrial DNA (mtDNA) that encode core subunits of electron transport chain complexes [1]. While converting energy to ATP and carrying out biosynthesis, mitochondria also generate free radicals that can damage DNA, proteins and lipids nearby [2]. Mitochondrial genome has no histone protection and lacks homologues recombination or other efficient repair mechanisms. As a result, mtDNA is particularly prone to accumulating mutations [3]. To make matter worse, inefficient electron transport chain (ETC) complexes caused by mtDNA mutations generate more free radicals and exacerbate the mitochondrial damage in a feed-forward cycle [2]. Accumulation of mtDNA mutations during lifetime has been postulated to cause age-related decline of energy metabolism and impairment of tissue homeostasis [4,5]. Mitochondrial “mutator” mice with an elevated rate of mtDNA mutagenesis display premature aging, which, in principle, substantiates the correlation between mtDNA mutations and aging [6,7]. However, mtDNA mutations from various tissues of normally aged human or experimental animals are found to be too low to possibly elicit any pathological consequences [8], which argues against a causative role of mtDNA mutations in physiological aging, particularly in the post mitotic tissues [9]. 

DNA replication is the source of mutations [10]. In adulthood, most tissues consist of post mitotic cells that have a slow turnover rate of mitochondria and mtDNA [11], which might explain the low mtDNA mutations frequency in post mitotic tissues. Therefore, the quest for connection between mtDNA mutations and aging might have focused on the wrong target from the very beginning. On the other hand, one would expect that mtDNA mutations in actively dividing cells, such as cancer cells and stem cells, could reach a high level during the aging process [12]. In fact, there is increasing evidence demonstrating the accumulation of mtDNA mutations in aged stem cells [13,14,15,16]. Stem cells are essential for tissue homeostasis and wound repair. Age dependent deterioration of stem cells contributes to several hallmarks of aging such as impaired capability of tissue repair and increased susceptibility to cancers and infectious diseases, and thereby has been proposed to play an important role in the natural aging process [17]. Stem cells in a quiescent state often emphasize glycolysis, presumably to maintain the genomic integrity by minimizing the production of damaging free radicals [18]. During active proliferation, stem cells undergo a metabolic reprograming to activate mitochondrial respiration, which is critical for their progenitors differentiation [18,19]. In principle, accumulation of mtDNA mutations in stem cells could cause age dependent deterioration of stem cells’ activity and competence. Nonetheless, the interplay between mtDNA mutations and stem cell aging remains to be explored. Besides the chronological aging, stem cells are also subject to proliferative aging, the accumulation of division cycles [20]. The continuous proliferation of stem cells would lead to not only the accumulation of damaged mutations, but also potentially the shortening of telomeres [21]. Telomere shortening has been observed in various types of aged stem cells in mammals, and hence ascribed as a factor contributing to the age associated decline of proliferation competency of stem cells [20]. Despite the progress in the field, our knowledge on how aging impacts stem cell functions remains limited. The cellular deficiencies caused by accumulation of proliferative cycles that contribute to the decline of stem cells’ activities remain to be defined. 

*Drosophila* oogenesis represents one of the best-characterized systems to study the behaviors of stem cells and the development of their progenitors. Germline stem cell (GSC) maintenance and division are regulated by bone morphogenic protein (BMP) signals from the surrounding niche [22]. The asymmetric division of GSC renews itself and generates a cystoblast, which continues to divide an additional four rounds with incomplete cytokinesis, generating 16 interconnected germ cells. One of them will develop into the eventual oocyte, while the remaining 15 become nurse cells. Over the adult life span, the decline of BMP signals in aged ovaries causes reduced proliferation and a gradual loss of the stem cell population [23]. About 70% of GSCs remain in two-month-old ovaries, and 40% of these are actively proliferating [23]. However, most female flies cease to lay eggs 40 days after eclosion, despite having viable GSCs [24]. As female flies age, there is a gradual reduction of hatching rate and increased incidences of developmental abnormalities of their eggs [25]. These observations suggest that some undefined deficiencies must have occurred in germ cells of aged female flies and impede the oogenesis and the development of their progeny. 

In current study, we utilized a physiological approach to manipulate GSC division cycle independent of their chronological age, and examine its impact on GSC aging and female reproductive physiology. We demonstrated that the accumulation of division cycles played a major role in maternal age dependent decline of eggs’ fitness and contributed to the age dependent decline of female fecundity. We also found that accumulation of division cycles led to a dramatic decline of cytochrome C oxidase activity, but did not cause telomere shortening in aged ovaries. Additionally, we detected increased mutations on mtDNA and observed impaired mtDNA replication in aged ovaries. The strong correlation between the decline of stem cell activity and mitochondrial dysfunction in aged ovaries, suggests that mtDNA mutations caused by proliferative cycles may contribute to stem cell aging.

## 2. Materials and Methods 

### 2.1. Fly Maintenance, Fecundity and Egg Hatching Assay

All flies were cultured with molasses corn meal food at 25 °C. For the fecundity assay, newly eclosed female flies were collected and cultured in vials with yeast. Each vial contained five female flies. The control group, two male flies, were put in the vials from the first day. The experimental groups were kept as virgin until the day 14 when male flies were introduced. Males used for the crosses were 2–3 days old and old males were replaced with young ones every three days. All flies were transferred to new food every day. Eggs laid in the vials were counted and kept at 25 °C until they hatched. Egg hatching rate was calculated as the total number of flies eclosed from the eggs normalized by the number of total eggs laid.

### 2.2. Mitochondrial Activity Staining on Ovaries

Histochemistry assays were performed according to a previous study [14], with some modifications. Briefly, ovaries were first incubated in cytochrome c oxidase assay medium (100 µM cytochrome c, 4 mM diaminobenzidine tetrahydrochloride, and 20 µg/mL catalase in 0.2 M phosphate buffer, pH 7.0) at room temperature for 30 min then 37 °C for 30 min. Ovaries were then washed in phosphate buffered saline (PBS) for three times and incubated in succinate dehydrogenase (SDH) assay medium (130 mM sodium succinate, 200 µM phenazine methosulphate, 1 mM sodium azide, 1.5 mM nitroblue tetrazolium in 0.2 M phosphate buffer, pH 7.0) at room temperature for 10 min. After washing in PBS, pH 7.4 for three times, ovaries were dehydrated in a graded ethanol series (70%, 95%, 2 × 100%), cleared in Histoclear (National Diagnostics, Atlanta, GA, USA) for 15 min, and mounted in DPX (VWR, Radnor, PA, USA) overnight.

### 2.3. Selection for mtDNA Mutations

The selection for mtDNA mutant flies was carried out as described previously [26]. Virgins of nanos-gal4 (Bloomington Stock #4937) were crossed with *UASp-MitoXhoI* males and the flies were transferred to a new bottle a week later for four consecutive weeks. The F1 female flies (*UASp-MitoXhoI/+; nanosgal4/+*) from each bottle representing different ages of F0 female were collected and mated individually (*n* > 100) with *w^1118^* males and the numbers of fertile females from each group were scored.

### 2.4. Quantitative Polymerase Chain Reaction Analysis of Telomere Length

Newly elcosed female flies were collected and kept in vials at 25 °C with or without male flies. After 1 day and 2 months, these female flies were dissected in PBS and ovaries were taken out for DNA extraction. The oligos for telomeric transposons *HeT-A*, *TAHRE*, *TART-A1* and a non-telomeric transposon *Jockey* were designed according to a previous study [27], and quantitative polymerase chain reaction (qPCR) was also carried out accordingly to the same study.

### 2.5. Immunofluorescence Staining, EdU Staining and In Situ Hybridization

5-Ethynyl-2’-deoxyuridine (EdU) incorporation assay was carried out as described previously [28]. The ovaries were first incubated with Schneider’s medium (Invitrogen, Carlsbad, CA, USA) containing 10% fetal bovine serum (Invitrogen) and 7 µM aphidicolin (Sigma-Aldrich, St. Louis, MO, USA) for 3 h at room temperature, followed by additional 2 h incubation with 10 µM EdU (Invitrogen) in the same medium. A Click-iT^®^ EdU Alexa Fluor^®^ 488 Imaging Kit (Invitrogen) was used to label the EdU. For the immunofluorescence staining of the germ line, cell division assay and EdU incorporation assay, the ovaries were fixed with 4% paraformaldehyde (PFA) in PBS, washed in PBS containing 0.1% Triton X-100, blocked with 5% bovine serum albumin (Sigma-Aldrich) or 5% goat serum (Invitrogen), and then stained with different antibodies or markers. 

For the in situ hybridization, the ovaries were fixed and prepared as described previously [29]. Same primer pairs used in real-time PCR assay were used to amplify the DNA fragments used for telemetric DNA probes. Primer pair His4-F1: 5′-TCCAAGGTATCACGAAGCC-3′ and His4-R1: 5′-AACCTTCAGAACGCCAC3′ was used for amplifying the gene his4 fragments for nuclear DNA probe. Ulysis™ Alexa Fluor^®^ 488 Nucleic Acid Labeling Kit (Invitrogen) was used to label the DNA probes. Ovaries were pretreated in 2.5 N HCl, 0.1% Triton X-100 for 5 min to denature DNA, and rinsed twice for 5 min in 0.1M NaBO_2_. Hybridization was performed at 37 °C in a hybridization buffer (5× saline sodium citrate, 40% HCONH_2_, 100 mg/mL *Escherichia coli* transfer RNA, 50 mg/mL heparin, 0.1% Triton X-100, 5 ng of probe/mL) overnight. 

The antibodies and reagents used were: rabbit anti-phospho-histone H3 (Millipore, ON, Canada, 06-570); rat anti-vasa (Developmental Studies Hybridoma Bank, Iowa city, IA, USA); Alexa Fluor secondary antibodies (Invitrogen) and Vectashield mounting medium with 4’,6-diamidino-2-phenylindole (DAPI) (Vector Laboratories, Burlingame, CA, USA). Image analysis was performed using the Carl Zeiss AxioObserver (Thornwood, NY, USA) equipped with PerkinElmer spinning disk confocal system (Waltham, MA, USA).

### 2.6. Rolling Cycle Amplification and Restriction Fragment Length Polymorphism analysis of mtDNA

**Figure 1:**
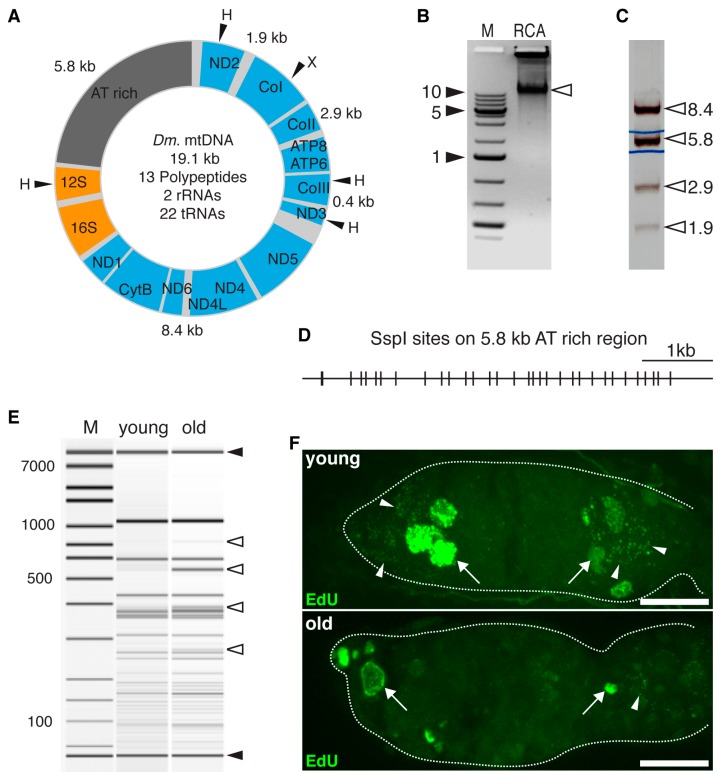
Mitochondrial DNA (mtDNA) rearrangements and impaired mtDNA replication in ovaries of aged flies. (**A**) Schematic drawing of *Drosophila melanogaster* (Dm.) mtDNA. Enzyme sites of HindIII (H) and XhoI (X) are indicated. Sizes of digested fragments are also labelled. (**B**) Representative gel image of rolling cycle amplification of mtDNA (arrowhead) and DNA marker (M, kb). (**C**) Pattern of rolling cycle amplification (RCA) amplified mtDNA digested by XhoI and HindIII. The 5.8 kb fragment spanning the AT-rich region was recovered for restriction fragment length polymorphism (RFLP) analysis. (**D**) Schematic map of SspI site on 5.8 kb AT-rich region. (**E**) densitometry plot of SspI digestion pattern of 5.8 fragments spanning the AT-rich region from young (2-day-old) and old (60-day-old) ovaries, analyzed by Agilent Bioanalyzer 2100. Note the difference of bands (open arrowheads) demonstrating the rearrangements of AT-rich regions in aged ovaries. DNA dye standard (closed arrowheads) and DNA ladder are marked (M, bp). (**F**) Representative images showing 5-ethynyl-2’-deoxyuridine (EdU) incorporation into mtDNA (green puncta, arrowheads) in ovaries (dashed line, anterior toward left) from young (2-day-old) and old (40-day-old) female flies. Note that the number of EdU puncta was dramatically reduced in germarium from old fly. Arrowhead: mtDNA; arrow: nuclear DNA; scale bar: 10 µm.

Mitochondrial DNA was amplified from total DNA preparations from ovaries of young or old female flies, through the rolling cycle amplification (RCA) amplification method. Briefly, 5 µL 10X Phi29 buffer (New England Biolab, Ipswich, MA, USA), 20 µL 2.5 mM dNTP and 23 µL mix of set of mtDNA specific oligos that span whole mtDNA described in a previously study [30], at final concentration of 1 mM each were mixed well, heated at 95 °C for 3 min and let cooled down at room temperature for another 5 min. Then 1 µL Bovine serum albumin and 1 µL Phi29 enzyme (New England Biolab, Ipswich, MA, USA) were added to the mix and incubated at 37 °C overnight. The RCA amplification usually produced a band at 20 kb and a large amount of amplified DNA stuck in the well (Figure 1B). Amplification mix was extracted by phenol/chloroform to remove the proteins and concentrated with ethanol precipitation. Precipitated RCA products were digested with XhoI and HindIII, which have one and four sites on mtDNA, respectively (Figure 1A). Since we were using total DNA as template, nuclear DNA was also amplified in RCA reaction. To remove the nuclear DNA contamination, a small amount of Acc651, BamHI, NcoI, SalI and SpeI that do not cut mtDNA were also included in the digestion mix. In addition, 5.8 kb fragment was recovered, further digested with SspI overnight and analyzed on BioAnalyzer 2100 (Agilent, Savage, MD, USA) using a High Sensitivity DNA Kit (Agilent) following the manufacturer’s protocol.

## 3. Results

### 3.1. Accumulation of Proliferation Cycles in Germline Stem Cells Causes Age Dependent Decline of Female Fecundity and Reduced Fitness of Their Progeny

**Figure 2:**
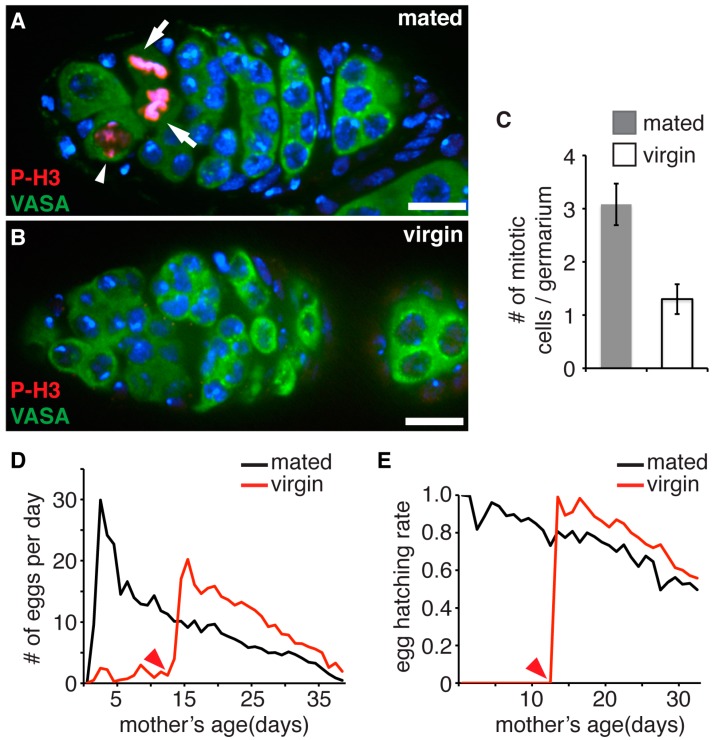
Germline stem cell (GSC) proliferation cycle contributes to the age dependent decline of female fecundity and progeny’s fitness. (**A**,**B**). Representative images of germarium from 1-week-old mated (**A**) or virgin (**B**) female, stained with anti-VASA (green) for germ line cells and anti- phosphorylated histone H3 (P-H3, red) for mitotic cells. In mated flies, dividing GSC (arrowhead) and germ cell cysts (arrows) are present. Scale bar: 10 µm. (**C**) Quantification of mitotic germ cells in germaria of mated and virgin female flies. No. (#) of mitotic germ cells flies is significantly less in virgin than mated flies (*n* = 100 germaria, *p* < 0.001). We also counted the P-H3 positive GSCs. There were 13 dividing GSCs in total from 100 germaria of mated female flies, but only four out of 100 virgin females. (**D**) Female fecundity was assayed by calculating the average egg production per female per day over 40 days. (**E**) Egg hatching rates plotted over maternal age. Black line, mated female flies; red line, female flies maintained as virgins for two weeks after eclosion. A total of 150 female flies were used for each group in (**D**,**E**). Each group was assayed in 30 vials with five female flies in each vial. Arrowhead, time point when virgin flies were mated with males.

We attempted to assess the impact of division cycles on GSCs using a physiological approach to manipulate their proliferation rate. During the normal mating process, males transfer sperm and seminal proteins, which simulate the ovulation and egg laying [31]. Keeping females as virgins slows down the oogenesis and the proliferation of germ cells including stem cells (Figure 2A–C), and therefore can be used to preserve the proliferative cycles of GSCs. We first collected virgin flies and divided them into two groups: the control group of flies were mated with wild type male flies immediately and the experimental group was maintained as virgins for two weeks before being mated with males. Fecundity of each group was scored by daily counting the number of eggs laid. Consistent with previous observations [24,25], the mated flies displayed the highest egg production during the first week. Then, their egg production gradually declined until the cessation of egg laying around 40-day-old (Figure 2D). During the first two weeks, the unmated females in the experimental group laid a small amount of unfertilized eggs (Figure 2D,E). After the onset of mating at day 15, they also showed a period of burst of egg production followed by a gradual decline of egg production as they aged. Significantly, at any given time point after the mating, the flies in the experimental group always laid more eggs than those in the control group (Figure 2D). Since control and experimental flies were siblings reared under identical culture conditions, the only difference in the two groups was that experimental flies were prevented from mating for two weeks, and thus underwent fewer GSCs replication cycles. This result demonstrates that, in addition to the physiological age, the accumulation of proliferation cycles undergone by GSCs contributes to the decline of their activity. We also examined the hatching rate of the eggs produced by each group of flies throughout their lifetime. We found that eggs produced by flies in the experimental group always hatched at a higher rate than those laid by control flies at any given time point after two weeks (Figure 2E). Interestingly, the hatching rates of progeny of the experimental group was very close to those produced by control flies two weeks earlier, suggesting that the replication cycle causes some uncharacterized cellular stresses in oocytes, which subsequently impair embryonic development.

### 3.2. Aging Does Not Lead to Telomere Shortening in Germline Stem Cells

**Figure 3:**
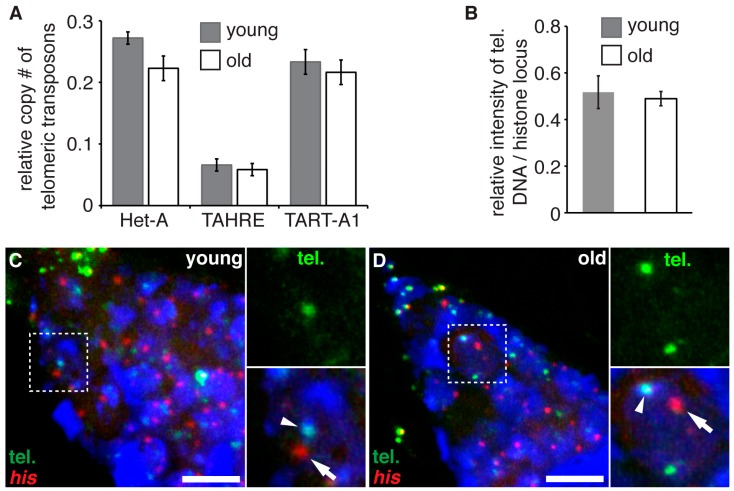
Aging does not lead to telomere shortening in GSCs. (**A**) Total DNA from the ovaries of young (2-day-old) and old (60-day-old) females were prepared and the copy numbers of three telomeric transposons *Het-A*, *TARAE* and *TART-A1* were determined by real time PCR and plotted (*n* = 3). A non-telomeric transposon *Joecy* was used as an internal control. (**B**) Quantifications of telomere (tel.) DNA contents by normalizing fluorescence in situ hybridization (FISH) signal of telomere DNA with FISH signal of his4 locus in GSCs of young and old females. No significant difference between young and old GSCs (*n* = 100 for each group). (**C**,**D**) Representative images of two color FISH against telomere DNA (green, mix of three probes against all three telomeric transposons) and *his4* locus (red) in young ((**C**), 2-day-old) and old ((**D**), 60-day-old) germaria. Box regions were enlarged for better illustrations of telomere DNA (arrowheads) and *his4* locus (arrows). Scale bar: 5 µm.

Continual division of stem cells could potentially lead to the gradual shortening of telomeres and the eventual replicative senescence [21]. *Drosophila* telomeres consist of a repetitive array of retrotransposons at the ends of each chromosome: *Het-A*, *TAHRE* and *TART-A1* [27]. It is not clear how aging might afflict telomere in *Drosophila* stem cells. We directly evaluated the impact of age on the telomere length of GSCs by quantifying the copy numbers of telomeric transposons in young and old ovaries. We found that the copy number of *Het-A* element was only slightly reduced in old ovaries, while there was no difference for either *TAHRE* or *TARTA1* between young and old ovaries (Figure 3A). We also directly visualized telomere DNA by fluorescence in situ hybridization (FISH) in GSCs (Figure 3C,D), and quantified their level by measuring the fluorescence intensity using the histone 4 locus as an internal control. Again, we found no difference of telomere DNA level between young and old GSCs (Figure 3B) in the in situ assay. Of note, telomere length does not appear to positively correlate with the female fecundity. Instead, increased telomere length actually decreases female fecundity [27]. Taken together, our data shows that the aging of GSCs does not result in significant telomere shortening, which seems to be a major factor causing the age dependent decline of GSCs activities.

### 3.3. Aged GSCs Have Increased mtDNA Mutations Loads and Defective Electron Chain Complexes

**Figure 4:**
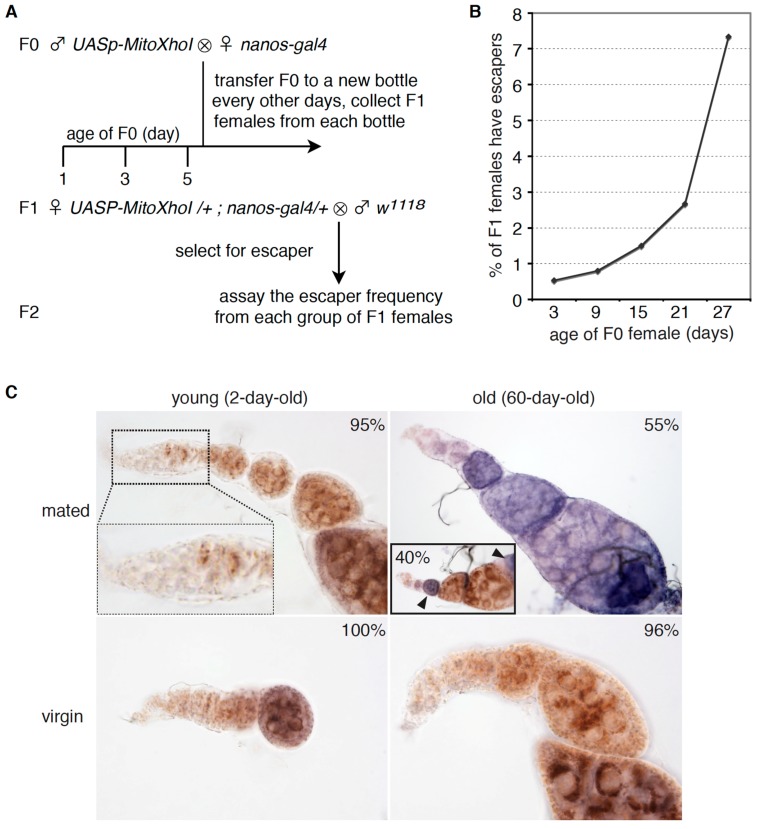
Aged GSCs have increased mtDNA mutation loads and defective electron transport chain complexes. (**A**) Cross scheme to assay the influence of maternal age (F0) on mtDNA mutation frequency in progeny (F1), as an indication of mtDNA mutation loads in F0 GSCs. (**B**) The different groups of F1 females expressing mitoXhoI in germ cells (*UASp-MitoXhoI/+; nanos-gal4/+, n* > 100) were produced by the same group of F0 females (*nanos-gal4*) at different ages. The frequency of fertile F1 females, as an indication of mtDNA mutation frequency in F0 GSCs is plotted against age of F0 females. The positive correlation between the number of fertile F1 females and the age of F0 females suggests that there were more mtDNA mutations in older GSCs in F0 females. (**C**) Ovaries of young (2-day-old) or old (60-day-old) females that were either mated with males or maintained as virgins were stained for dual succinate dehydrogenase and cytochrome C oxidase (COX) activity. The representative images and their percentage (*n* = 20) for each group are shown. Nearly 100% of ovaries of young mated females, young virgins and old virgins displayed normal COX activity (brown color). Note that the anterior part of the germarium (boxed and enlarged view), the location of stem cells and early stage cysts are nearly colorless, showing that mitochondria are mostly inactive in this developmental stage. In addition, 55% of ovaries from old mated females were completely lacking in COX activity (blue color). Forty percent of ovaries from old mated flies (inserted panel) showed a mosaic pattern of normal and deficient COX activity (arrowheads), suggesting that COX is normal in one germ line stem cell, but disrupted in another one in the same ovariole.

An unavoidable consequence of continual proliferation of stem cells is the accumulation of damaging mutations on both nuclear and mitochondrial genomes, which could impair the survival, proliferation and differentiation of stem cells. Mitochondrial DNA is particularly prone to mutation accumulation due to the lack of recombination or other effective repair mechanisms. Indeed, the mutation frequency has been estimated to be 10 s to 100 folds higher on mtDNA than nuclear genome [3]. We applied a selection scheme based on a mitochondrially targeted restriction enzyme, MitoXhoI, to indirectly evaluate mtDNA mutation frequency in GSCs [26]. Expression of MitoXhoI in female GSCs leads to the selection of rare escapers carrying mtDNA mutations that disrupt the single XhoI site on mtDNA. Thus, the frequency of escapers surviving through the selection would correlate with mtDNA mutation load in GSCs, i.e., the higher the mutation load, the more escapers selected. Based on this reasoning and the rule of maternal inheritance for mtDNA, we designed a scheme to quantify escapers produced by F1 females (*UASp-MitoXhoI; nanosgal4*), which were progeny of the same group of F0 female flies (nanosgal4) at different ages, ranging from one day to four weeks (Figure 4A,B). We expected that F0 females at old age would deposit more mtDNA mutations to F1 females, which, thereby, would lead to more grandchildren escapers. Approximately 1% of the F1 females produced by 1~2-day-old grandmothers (F0) gave rise to escapers. The frequency dramatically increased according to the age of grandmothers (Figure 4B). Nearly 10% of F1 female progenies produced by 4-week-old F0 flies had escapers. F1 females from different bottles were cultured under identical conditions and inherited mtDNA from the same pool of GSCs of grandmothers (F0). The higher frequency of grandchildren escapers (F2) that derived from older F0 flies suggested that older GSCs had a higher mtDNA mutations loads. 

Mitochondrial DNA encodes essential subunits of the electron transport chain that drives ATP synthesis to fuel virtually every single cellular process. Each cell contains 100–1000 copies of mtDNA. Mitochondrial DNA mutations at a low level could be complemented by the wild type genome in the same cell and thereby might not elicit any phenotypic consequences. Next, we asked whether mitochondrial activities were impaired in aged GSCs. We stained for activities of mitochondrial complex II and IV in young and old ovaries. In this colorimetric assay, the brown color indicates activity of both complex II and IV, while the blue color indicates complex II activity in the absence of complex IV activity [13,32]. The young ovaries displayed a brown color, demonstrating that both complex II and IV are active. However, 60% of ovaries from 63-day-old females displayed a blue color, indicating the presence of active complex II, but reduced level or absence of complex IV activity (Figure 4C). Complex II consists of four subunits, all of which are encoded in nuclear genome. On the contrary, three core components of a total of 10 subunits of complex IV are encoded by mitochondrial DNA. Defective complex IV, but normal complex II is considered as a hallmark of mtDNA defects [32]. These results suggest that there are mtDNA lesions in aged ovaries while the nuclear genome appears intact. These mtDNA lesions could block the mtDNA replication, transcription, or directly disrupt protein activity. To further test whether the replication cycle contributes to this age dependent occurrence of mitochondrial dysfunction, we assayed complexes’ activities in ovaries of 60-day-old virgin flies. Strikingly, 95% of ovaries of two-month-old virgin females displayed active complex II and IV (Figure 4C). Taken together, these results support that the continual proliferation of GSCs leads to increased mtDNA mutations, which may impair mitochondrial activity, germ cell proliferation and development.

### 3.4. Aged Germline Stem Cells Have Rearrangements on mtDNA AT-Rich Region and Impaired mtDNA Replication

To directly measure the frequency and understand the nature of mtDNA mutations, we applied deep sequencing on full-length mtDNA that was amplified by RCA. However, the mutation frequencies from both samples were around 1.0%, similar to the background error rate of the procedure [33], and thereby precluding further analysis (data not shown). Additionally, deep sequencing also failed to cover the AT-rich region on mtDNA, which consists of multiple repetitive elements ([34] and Figure 1A). AT-rich regions are present on mtDNA of all *Drosophila* species, but their lengths and organizations are highly variable [34], while the coding regions are much more conserved. In addition, a deletion in AT-rich region is readily emerged in a lab culture over the span of 10 generations [35]. These observations suggest that AT-rich regions are highly mutable, and therefore constitute a sensitive target for assaying mtDNA mutations. To analyze the potential mutations or DNA rearrangements, we recovered an AT-rich region from an RCA product and assayed its integrity with RFLP analysis using restriction enzyme SspI (Figure 1B–D). We found there are several different bands between AT-rich region fragments from young and old ovaries, demonstrating the presence of rearrangements or mutations on the AT-rich region of mtDNA from old ovaries (Figure 1E). *Drosophila* mtDNA replication origin is located in the AT-rich region [34]. We reasoned that these mutations on AT-rich regions of old GSCs could interfere with the mtDNA replication. To directly test this idea, we labeled mtDNA replication using EdU incorporation in young and aged ovaries. We found that mtDNA incorporations were evident in the anterior tip of the germarium, where GSCs are located, and at the late stage of the germarium (Figure 1F). However, there were very few EdU puncta in cytoplasm of 60-day-old ovaries, while nuclear DNA incorporation was still present (Figure 1F). It demonstrated that germ cells in old ovaries were still capable of carrying out nuDNA replication, but were specifically deficient in mtDNA replication. Taken together, these results suggest the mutations on AT-rich regions likely impair mtDNA replication in aged GSCs and their progenitors.

## 4. Discussion

We demonstrated that the division cycle of GSCs, but not solely the chronological age, contributes to the age dependent decline of GSCs function, which is likely to be the major causative factor of the reduced hatching rate of embryos produced by old mothers. We also noticed that accumulation of proliferation cycles led to increased mtDNA mutation load in aged GSCs based on two indirect approaches: mtDNA mutations selection and RFLP analysis of AT-rich region. However, neither of these assays provided a quantitative measure for mutation frequency. Also, it is puzzling that we did not detect an obvious increase of mutation load in aged ovaries via mtDNA deep sequencing. It has been documented that more than 60% of mutation load, corresponding to a mutation frequency around 10^−4^ per nucleotide is required to elicit any discernible phenotype in cultured cells [8]. It is possible that the actual mutation rate might be below the systematic error rate at 1% [33], but higher than 10^−4^ in aged ovaries, which could disrupt ETC complexes or impede mtDNA replication as demonstrated in aged ovaries. Alternatively, the observed 10-fold increase of escapers carrying mtDNA mutations by old mothers might be simply due to the continual proliferation of few GSCs carrying high level of mtDNA mutations. Interestingly, a mosaic pattern of the ETC activity staining was observed in a few aged ovaries, in which some germ cells had normal mitochondrial activity, while others were clearly defective in complex IV (Figure 4C, insert). This resembles the clonal expansions of mtDNA mutations that have been demonstrated in human tissues and cells [13,36]. Ideally, more elegant assays such as laser microdissection or single cell sequencing [13,36], shall be applied to quantitatively assess mtDNA mutation frequency, which would help to resolve this discrepancy. Additionally, other age-associated deficiencies such as mtDNA depletion [37], and accumulation of oxidative damages [2], could also contribute to the mitochondrial deterioration in aging stem cells. Therefore, the data presented in this study should be interpreted with caution.

The AT-rich region seems to be particularly susceptible to mutations based on our data, which is consistent with previous studies [34,35]. Although mammalian mitochondrial genomes lack AT-rich regions, the presence of single strand DNA structure in the D-loop could render them vulnerable to mutations accumulation [38]. Thus, the impacts of the division cycle on stem cell function present a great challenge for long-lived mammals. Sophisticated mechanisms must be evolved to preserve the replication cycles of stem cells in order to maintain their fitness over a long lifespan. For example, in the process of mammalian hematopoiesis, the multipotent hematopoietic stem cell normally divides infrequently to produce committed progenitor cells, which divide multiple rounds before differentiating into mature blood cells [39]. It has been proposed that the amplifying divisions of committed progenitors help to reduce the risk of replicative senescence caused by telomere shortening [39]. However, rodents have active telomerase and rather long telomeres in somatic tissues [40], yet still employ the same mechanism to reduce the replication cycles of stem cells. This argues against the idea that the risk of telomere shortening is the major evolution pressure underlying this conserved aspect of hematopoiesis in all mammals. In current study, we did not find significant telomere shortening in aged GSCs. Instead, continual division of GSCs led to the accumulation of mtDNA deficiencies, which could potentially disrupt mitochondrial respiration complexes and likely contributed to the age dependent decline of GSC activity as demonstrated by gradual decline of female fecundity. 

In most long-lived post-mitotic tissues, mitochondria undergo continual proliferation, while the nuclear genome becomes quiescent [11]. Age dependent accumulation of mtDNA mutations has been found in various tissues of experimental animals and humans, and thereby has been proposed to play a causative role in aging [41]. However, mitochondrial mutations in most aged post-mitotic tissues are too low to cause any functional consequences [8,9], as mitochondria of aged tissues remain biochemically active [8,9]. It is confounding why the replication cycle of mtDNA only causes mutation accumulation in stem cells, but not in post-mitotic cells. It has been proposed that continual mitochondrial biogenesis and turnover in post-mitotic cells underlie a quality control mechanism to weed out defective mitochondria [42]. Recent studies suggest that the autophagy pathway might be required to degrade defective mitochondria with low inner membrane potential, the electrochemical potential generated through active mitochondria respiration [43]. Though the detailed mechanism of mitochondrial quality control remains to be fully elucidated, it is a reasonable assumption that active mitochondrial respiration is a prerequisite for a cell to distinguish healthy vs. defective mitochondria. Drosophila female GSCs have been suggested to emphasize glycolysis instead of oxidative phosphorylation to produce ATP [44]. Mitochondrial enzymatic staining also confirmed that electron transport chain complexes in early-stage GSCs in the germarium were inactive (Figure 4C), which may render GSCs incapable of recognizing mitochondria carrying mtDNA mutations. We propose that stem cells might be particularly prone to accumulation of mtDNA mutations due to two factors: i) continual replications that lead to mutation accumulation and ii) the lack of efficient quality control mechanism against defective mitochondria. It has been well documented that mitochondria are inactive in a few types of mammalian stem cells [45,46,47,48]. In addition, mtDNA mutations have been identified in mouse and human stem cells and have been suggested to play a causal role in stem cell aging [13,16]. Therefore, the replication cycle and the accumulation of mtDNA mutations might be a conserved mechanism underlying stem cell aging in metazoans. 

## References

[1] Wallace D.C. (2008). Mitochondria as chi. Genetics.

[2] Balaban R.S., Nemoto S., Finkel T. (2005). Mitochondria, oxidants, and aging. Cell.

[3] Pesole G., Gissi C., De Chirico A., Saccone C. (1999). Nucleotide substitution rate of mammalian mitochondrial genomes. J. Mol. Evol..

[4] Krishnan K.J., Greaves L.C., Reeve A.K., Turnbull D. (2007). The ageing mitochondrial genome. Nucleic Acids Res..

[5] Linnane A.W., Marzuki S., Ozawa T., Tanaka M. (1989). Mitochondrial DNA mutations as an important contributor to ageing and degenerative diseases. Lancet.

[6] Kujoth G.C., Hiona A., Pugh T.D., Someya S., Panzer K., Wohlgemuth S.E., Hofer T., Seo A.Y., Sullivan R., Jobling W.A. (2005). Mitochondrial DNA mutations, oxidative stress, and apoptosis in mammalian aging. Science.

[7] Trifunovic A., Wredenberg A., Falkenberg M., Spelbrink J.N., Rovio A.T., Bruder C.E., Gidlöf S., Oldfors A., Wibom R., Törnell J. (2004). Premature ageing in mice expressing defective mitochondrial DNA polymerase. Nature.

[8] Lightowlers R.N., Jacobs H.T., Kajander O.A. (1999). Mitochondrial DNA—All things bad?. Trends Genet..

[9] Khrapko K., Vijg J. (2007). Mitochondrial DNA mutations and aging: A case closed?. Nat. Genet..

[10] Larsson N.-G. (2010). Somatic mitochondrial DNA mutations in mammalian aging. Annu. Rev. Biochem..

[11] Menzies R.A., Gold P.H. (1971). The turnover of mitochondria in a variety of tissues of young adult and aged rats. J. Biol. Chem..

[12] Baines H.L., Turnbull D.M., Greaves L.C. (2014). Human stem cell aging: Do mitochondrial DNA mutations have a causal role?. Aging Cell.

[13] Taylor R.W., Barron M.J., Borthwick G.M., Gospel A., Chinnery P.F., Samuels D.C., Taylor G.A., Plusa S.M., Needham S.J., Greaves L.C. (2003). Mitochondrial DNA mutations in human colonic crypt stem cells. J. Clin. Investig..

[14] McDonald S.A.C., Greaves L.C., Gutierrez-Gonzalez L., Rodriguez-Justo M., Deheragoda M., Leedham S.J., Taylor R.W., Lee C.Y., Preston S.L., Lovell M. (2008). Mechanisms of field cancerization in the human stomach: The expansion and spread of mutated gastric stem cells. Gastroenterology.

[15] Fellous T.G., Islam S., Tadrous P.J., Elia G., Kocher H.M., Bhattacharya S., Mears L., Turnbull D.M., Taylor R.W., Greaves L.C. (2009). Locating the stem cell niche and tracing hepatocyte lineages in human liver. Hepatology.

[16] Ahlqvist K.J., Hämäläinen R.H., Yatsuga S., Uutela M., Terzioglu M., Götz A., Forsström S., Salven P., Angers-Loustau A., Kopra O.H. (2012). Somatic progenitor cell vulnerability to mitochondrial DNA mutagenesis underlies progeroid phenotypes in Polg mutator mice. Cell Metab..

[17] Ho A.D., Wagner W., Mahlknecht U. (2005). Stem cells and ageing. EMBO Rep..

[18] Folmes C.D.L., Nelson T.J., Dzeja P.P., Terzic A. (2012). Energy metabolism plasticity enables stemness programs. Ann. N.Y. Acad. Sci..

[19] Xu X., Duan S., Yi F., Ocampo A., Liu G.-H., Izpisua Belmonte J.C. (2013). Mitochondrial regulation in pluripotent stem cells. Cell Metab..

[20] Oh J., Lee Y.D., Wagers A.J. (2014). Stem cell aging: Mechanisms, regulators and therapeutic opportunities. Nat. Med..

[21] Günes C., Rudolph K.L. (2013). The role of telomeres in stem cells and cancer. Cell.

[22] Drummond-Barbosa D. (2008). Stem cells, their niches and the systemic environment: An aging network. Genetics.

[23] Pan L., Chen S., Weng C., Call G., Zhu D., Tang H., Zhang N., Xie T. (2007). Stem cell aging is controlled both intrinsically and extrinsically in the *Drosophila* ovary. Cell Stem Cell.

[24] Waskar M., Li Y., Tower J. (2005). Stem cell aging in the *Drosophila* ovary. Age.

[25] Zhao R., Xuan Y., Li X., Xi R. (2008). Age-related changes of germline stem cell activity, niche signaling activity and egg production in *Drosophila*. Aging Cell.

[26] Xu H., DeLuca S.Z., O’Farrell P.H. (2008). Manipulating the metazoan mitochondrial genome with targeted restriction enzymes. Science.

[27] Walter M.F., Biessmann M.R., Benitez C., Török T., Mason J.M., Biessmann H. (2007). Effects of telomere length in *Drosophila melanogaster* on life span, fecundity and fertility. Chromosoma.

[28] Hill J.H., Chen Z., Xu H. (2014). Selective propagation of functional mitochondrial DNA during oogenesis restricts the transmission of a deleterious mitochondrial variant. Nat. Genet..

[29] Lécuyer E. (2011). High resolution fluorescent in situ hybridization in *Drosophila*. Methods Mol. Biol..

[30] Haag-Liautard C., Coffey N., Houle D., Lynch M., Charlesworth B., Keightley P.D. (2008). Direct estimation of the mitochondrial dna mutation rate in *Drosophila melanogaster*. PLoS Biol..

[31] Wolfner M.F. (2002). The gifts that keep on giving: Physiological functions and evolutionary dynamics of male seminal proteins in *Drosophila*. Heredity.

[32] Johnson M.A., Bindoff L.A., Turnbull D.M. (1993). Cytochrome c oxidase activity in single muscle fibers: Assay techniques and diagnostic applications. Ann. Neurol..

[33] Ni T., Wei G., Shen T., Han M., Lian Y., Fu H., Luo Y., Yang Y., Liu J., Wakabayashi Y. (2015). MitoRCA-seq reveals unbalanced cytocine to thymine transition in *Polg* mutant mice. Sci. Rep..

[34] Lewis D.L., Farr C.L., Kaguni L.S. (1995). *Drosophila melanogaster* mitochondrial DNA: Completion of the nucleotide sequence and evolutionary comparisons. Insect Mol. Biol..

[35] Rand D.M. (2011). Population genetics of the cytoplasm and the units of selection on mitochondrial DNA in *Drosophila melanogaster*. Genetica.

[36] Nekhaeva E., Bodyak N.D., Kraytsberg Y., McGrath S.B., Orsouw N.J.V., Pluzhnikov A., Wei J.Y., Vijg J., Khrapko K. (2002). Clonally expanded mtDNA point mutations are abundant in individual cells of human tissues. PNAS.

[37] May-Panloup P., Boucret L., Chao de la Barca J.M., Desquiret-Dumas V., Ferré-L’Hotellier V., Morinière C., Descamps P., Procaccio V., Reynier P. (2016). Ovarian ageing: the role of mitochondria in oocytes and follicles. Hum. Reprod. Update.

[38] Lee H.-C., Yin P.-H., Lin J.-C., Wu C.-C., Chen C.-Y., Wu C.-W., Chi C.-W., Tam T.-N., Wei Y.-H. (2005). Mitochondrial genome instability and mtDNA depletion in human cancers. Ann. N. Y. Acad. Sci..

[39] Alberts B., Johnson A., Lewis J., Raff M., Roberts K., Walter P. (2002). Molecular Biology of the Cell.

[40] Prowse K.R., Greider C.W. (1995). Developmental and tissue-specific regulation of mouse telomerase and telomere length. Proc. Natl. Acad. Sci. USA.

[41] Nagley P., Wei Y.-H. (1998). Ageing and mammalian mitochondrial genetics. Trends Genet..

[42] Tolkovsky A.M. (2009). Mitophagy. Biochim. Biophys. Acta.

[43] Jin S.M., Youle R.J. (2012). PINK1- and Parkin-mediated mitophagy at a glance. J. Cell Sci..

[44] Kai T., Williams D., Spradling A.C. (2005). The expression profile of purified *Drosophila* germline stem cells. Dev. Biol..

[45] Margineantu D.H., Hockenbery D.M. (2016). Mitochondrial functions in stem cells. Curr. Opin. Genet. Dev..

[46] Simsek T., Kocabas F., Zheng J., Deberardinis R.J., Mahmoud A.I., Olson E.N., Schneider J.W., Zhang C.C., Sadek H.A. (2010). The distinct metabolic profile of hematopoietic stem cells reflects their location in a hypoxic niche. Cell Stem Cell.

[47] Varum S., Rodrigues A.S., Moura M.B., Momcilovic O., Iv C.A.E., Ramalho-Santos J., Houten B.V., Schatten G. (2011). Energy metabolism in human pluripotent stem cells and their differentiated counterparts. PLoS ONE.

[48] Zhou W., Choi M., Margineantu D., Margaretha L., Hesson J., Cavanaugh C., Blau C.A., Horwitz M.S., Hockenbery D., Ware C. (2012). HIF1α induced switch from bivalent to exclusively glycolytic metabolism during ESC-to-EpiSC/hESC transition. EMBO J..

